# Characterizing interregional differences in the rheological properties and composition of rat small intestinal mucus

**DOI:** 10.1007/s13346-024-01574-1

**Published:** 2024-03-25

**Authors:** Mette Klitgaard, Jette Jacobsen, Maja Nørgaard Kristensen, Ragna Berthelsen, Anette Müllertz

**Affiliations:** Department of Pharmacy, University of Copenhagen, Universitetsparken 2, 2100 Copenhagen, Denmark

**Keywords:** Mucus pH, Bile salts, Lipids, Charged aerosol detection, Rheology

## Abstract

The mucus layer in the small intestine is generally regarded as a barrier to drug absorption. However, the mucus layer is a complex system, and presently, only a few studies have been conducted to elucidate its physicochemical properties. The current study hypothesizes that the mucus layer contains solubility-enhancing surfactants and thus might aid the oral absorption of poorly water-soluble drugs. Mucus was sampled from sections of the small intestine of fasted rats to analyze the rheological properties and determine the mucus pH and concentrations of proteins and endogenous surfactants, i.e., bile salts, polar lipids, and neutral lipids. The mucus layer in the two proximal sections of the small intestine exhibited different rheological properties such as higher zero-shear viscosity and lower loss tangent and higher protein concentrations compared to all subsequent sections of the small intestine. The pH of the mucus layer was stable at ~ 6.5 throughout most of the small intestine, but increased to 7.5 in the ileum. The bile salt concentrations increased from the duodenum (16.0 ± 2.2 mM) until the mid jejunum (55.1 ± 9.5 mM), whereas the concentrations of polar lipids and neutral lipids decreased from the duodenum (17.4 ± 2.2 mM and 37.8 ± 1.6 mM, respectively) until the ileum (4.8 ± 0.4 mM and 10.7 ± 1.1 mM, respectively). In conclusion, the mucus layer of the rat small intestine contains endogenous surfactants at levels that might benefit solubilization and absorption of orally administered poorly water-soluble drugs.

**Graphical Abstract:**

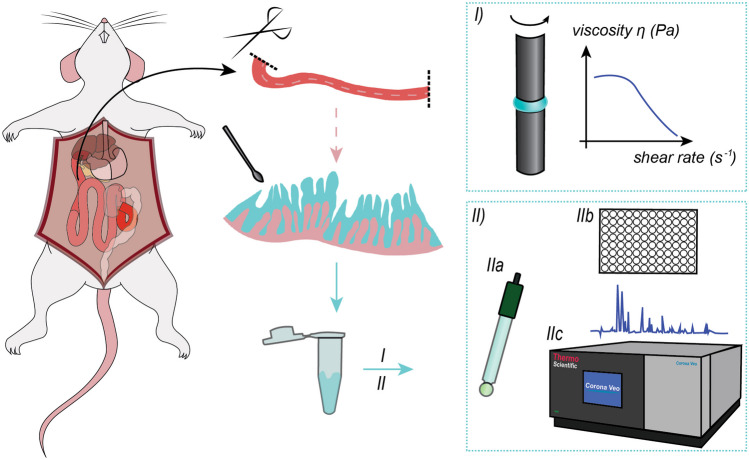

**Supplementary Information:**

The online version contains supplementary material available at 10.1007/s13346-024-01574-1.

## Introduction

Mucus is a complex, viscoelastic hydrogel lining various cavities and internal surfaces of the body. While the properties, composition, and function of mucus change depending on its location, it always consists of 95% water, 0.5-5% mucins, and smaller amounts of proteins, lipids, immunoglobulins, electrolytes, and cellular debris [1–3]. The mucus layer of the small intestine is most often seen as a protective barrier as well as a barrier for oral drug absorption [3, 4]. However, the mucus layer is still poorly understood, and only a few studies have thoroughly characterized the rheological and physicochemical properties of small intestinal mucus [5–11]. At present there is very limited information on human small intestinal mucus available in the literature, and the knowledge hereof is either extrapolated from other sources of mucus in the human body or from small intestinal mucus in animals [10, 12]. In vitro studies of oral drug absorption have often used dispersions of partially purified, commercially available mucins to simulate the mucus layer [1, 2]. Unfortunately, studies have shown that commercially available mucins, such as porcine gastric mucin, cannot replicate the properties of the native small intestinal mucus layer [5, 13]. This is, in part, thought to be due to the attributes of the non-mucin components of the mucus layer. In a study by Larhed et al., the composition of small intestinal porcine mucus was studied, and high levels of lipids (37% (w/w)) and proteins (39% (w/w)) were found in dried small intestinal mucus. The authors furthermore studied the diffusion of five model drugs (one hydrophilic and four lipophilic) through diffusion media containing the individual mucus components in comparison to diluted porcine mucus. They found that the barrier properties were highly dependent on the lipids present in the mucus [5]. Boegh et al. developed a biosimilar mucus based on the porcine mucus characteristics reported by Larhed et al. [5, 7]. Boegh et al. found that the rheological properties and mucus network could not be recreated by the commercially available porcine gastric mucin and artificial polymer alone since these properties also depend on the lipids of the mucus [7].

In vitro models assessing drug solubilization and absorption in the gastrointestinal tract of preclinical species display better predictability when carried out at conditions simulating the preclinical species in which the study was performed, compared to the conditions of humans [14–16]. As the rat is frequently used in preclinical oral drug delivery studies and studies have shown a good correlation between the absorption of orally administered drugs to humans and rats [17–19], it is practical to simulate the gastrointestinal conditions of the rat in vitro. However, to do this, the physiological conditions of the rat gastrointestinal tract, relevant for oral absorption of poorly water-soluble drugs, need to be assessed. A recent study by our group reported values for the luminal pH and concentrations of endogenous surfactants, i.e., bile salts, polar lipids, and neutral lipids, in rat gastrointestinal fluids, thus expanding the understanding of this preclinical species concerning oral drug delivery [20]. However, the rheological and physicochemical properties of the small intestinal mucus layer of the rat are still relatively unexplored. While the viscous mucus layer might slow the diffusion of orally administered drugs, it is hypothesized that it also contains solubilizing endogenous surfactants, such as lipids and bile salts, at levels that could facilitate the dissolution and absorption of poorly water-soluble drugs. Thus, the current study aimed to characterize the mucus layer in the small intestine of the fasted rat with regard to; rheological properties, pH and the concentrations of proteins, bile salts, polar lipids, and neutral lipids. As the current study was performed in parallel with the study by Klitgaard et al., the reported luminal values will be used for comparison [20]. Overall, the current study aimed to improve the understanding of the physicochemical properties of the mucus layer in the rat small intestine, thereby aiding the development of better in vitro models to study the effect of the mucus layer on oral drug absorption and eventually the development of better oral drug delivery systems.

## Materials and methods

### Materials

Acetonitrile, ammonium acetate, isopropanol, and methanol (> 99.9%) were all purchased from VWR Chemicals (Leuven, Belgium). Formic acid was obtained from Fischer Scientific (Waltham, MA, USA) and sodium taurocholate hydrate (96%) was purchased from Alfa Aesar (Kandel, Germany). Sodium glycocholate hydrate (> 98%) was bought from abcr (Karlsruhe, Germany) and lysophosphatidylcholine from soybean was generously donated from Lipoid (Ludwigshafen, Germany). Chenodeoxycholic acid (≥ 96%), cholesterol (≥ 99%), cholic acid (≥ 98%), hyodeoxycholic acid (≥ 98%), linoleic acid (≥ 99%), linolenic acid (≥ 99%), lysophosphatidylcholine from egg yolk (European Pharmacopeia reference standard), oleic acid (≥ 99%), palmitic acid (≥ 99%), phosphatidylcholine from egg yolk (European Pharmacopeia reference standard), phosphatidylcholine from soya bean (European Pharmacopeia reference standard), phosphatidylethanolamine from soya bean (European Pharmacopeia reference standard), sodium deoxycholate (≥ 98%), sodium glycochenodeoxycholate (≥ 97%), sodium glycodeoxycholate (≥ 97%), sodium taurochenodeoxycholate (≥ 95%), sodium taurodeoxycholate hydrate (≥ 95%), sphingomyelin from chicken egg yolk (≥ 98.0%), stearic acid (approx. 99%), tauro-α-muricholic acid sodium salt (> 99%), tauro-β-muricholic acid sodium salt (> 99%), ursodeoxycholic acid (≥ 99%), α-muricholic acid (> 99%), β-muricholic acid (≥ 98%), and ω-muricholic acid (> 99%) were purchased from Sigma-Aldrich (St. Louis, MO, USA). Purified water was prepared from a SG ultra-clear UV apparatus from Holm & Halby Service (Brøndby, Denmark).

### Animal study

The animal study was conducted at the University of Copenhagen under license number 2019-15-0201-00262 in compliance with the Danish laws on animal experiments, the EU Directive 2010/63/EU, and the ARRIVE guidelines [21].

Fourteen male Sprague-Dawley rats were purchased from Janvier Labs (Le Genest-Saint-Isle, France). All rats were kept in a room with a reversed day-night cycle (12/12 h, lights on at 20:00) and acclimatized for at least seven days before the experiment. The rats were fasted for 15-19 h before the experiments with *ad libitum* access to water. The average weight of the rats on the day of the experiments was 338 ± 8 g. The fasted rats were killed by carbon dioxide gas, and the small intestine was extracted *postmortem*. The small intestine was divided into sections of approx. 15 cm starting from the pylorus and ending at the ileocecal junction, producing eight sections in total (duodenum, proximal jejunum sections 1 and 2, mid jejunum sections 1 and 2, distal jejunum sections 1 and 2, and ileum). The mucus was gently scraped off each intestinal section using a laboratory spoon and transferred to polypropylene microcentrifuge tubes. For each rat, the mucus was isolated within 30 min *postmortem*. The mucus samples were divided into two groups to (i) determine the rheological properties of the mucus (*n* = 6) and (ii) determine the pH and to quantify concentration proteins and endogenous surfactants in the mucus (*n* = 8). For the latter group, the pH of each mucus section was measured three times at room temperature within 3–4 h after isolation using a micro pH electrode (Metrohm, Herisau, Switzerland) connected to a portable pH meter PHM201 (Radiometer Analytical, Lyon, France). All samples were stored at -18 °C until further analysis.

### Rheology

The rheological properties of the mucus from six rats were determined using an AR-G2 parallel-plate (8 mm diameter geometry, steel) and a rotational rheometer from TA Instruments (New Castle, DE, USA) with a gap of 500 µm. To minimize evaporation from the samples during testing, the analyses were not be performed at the physiologically relevant 37 °C, and the temperature was thus kept at 25.0 ± 0.3 °C in an environmental test chamber with pressured air. Similarly, the samples were loaded at a slight intentional overload (80 µL) to limit the effect of evaporation during the experiments. An initial stress sweep test (frequency 1 Hz, torque 3.0·10^-3^-2.0·10^4^ µN·m) was performed on pooled small intestinal mucus from a pilot study to determine a test torque in the linear viscoelastic region. For the actual samples, a frequency sweep (angular frequencies 0.1-100 rad/s, torque 8.0 µN·m) was followed by a flow sweep (shear rate 1.0·10^-3^-1.0·10^2^ s^-1^). TA Instruments TRIOS v3.3.1.4364 was used to assess and analyze the rheological data from the experiments. The software was used to determine the zero shear viscosity (through the Carreau-Yasuda model), the onset of shear-thinning (from the tangential intercept of the zero shear viscosity and the shear thinning region), and slope of shear thinning (shear rates 0.03-10 s^-1^) on the flow curves of the mucus from the different sections of the rat small intestine. The loss tangent, tan δ, was determined from the storage modulus G′ and loss modulus G″ from the frequency sweep at angular frequency 1 rad/s according to Eq. 1 to evaluate if the mucus gel showed predominantly solid-like (tan δ < 1) or liquid-like behavior (tan δ > 1) [10, 22, 23].

1 $$tan\delta =\frac{G"}{G^{\prime}}$$

### Quantification of proteins

The mucus protein content was determined from mucus samples of eight rats using a Pierce™ BCA Protein Assay Kit from Thermo Scientific (Waltham, MA, USA). Samples were diluted 200 times in saline and the protein content quantified according to the manufacturer’s specifications in a 96-well plate using a CLARIOstar^®^ *Plus* microplate reader from BMG Labtech (Ortenberg, Germany).

### Quantification of endogenous surfactants

The utilized quantification method is described in detail in Klitgaard et al. [20]. The mucus from eight rats was used to quantify endogenous surfactants, i.e., bile salts, polar lipids, and neutral lipids, by reverse-phase high-performance liquid chromatography with charged aerosol detection (RP-HPLC-CAD). In short, the mucus samples were weighed and diluted in cold methanol containing an internal standard mix to precipitate the proteins. Following centrifugation using a Heraeus Biofuge 15 centrifuge from Thermo Fisher Scientific (Osterode, Germany) at 13,300 rpm for 10 min (16,810 × g at r_max_), the supernatant was analyzed by RP-HPLC-CAD using a Dionex Ultimate 3000 pump, Dionex Ultimate 3000 Autosampler, Dionex Ultimate 3000 column compartment, and a Corona Veo Charged Aerosol Detector from Thermo Scientific (Waltham, MA, USA). The sample content was separated on an ACE Excel5 SuperC18 250 × 3.0 mm column with an ACE 5 SuperC18 analytical guard cartridge from Advanced Chromatography Technologies Ltd (Aberdeen, Great Britain) using a gradient of ammonium acetate buffer (pH 4), methanol, acetonitrile, and isopropanol within 60 min. Each peak was identified through comparison to pure standard solutions of representative bile salts, polar lipids, and neutral lipids.

### Statistics

Microsoft Excel 2016 (Redmond, WA, USA) was used to process the data. GraphPad Prism v. 9.3.1 (GraphPad Software Inc., San Diego, CA, USA) was used to visualize the data graphically and analyze it statistically. One-way ANOVA followed by a Tukey post hoc analysis was applied to analyze any statistical difference between the values of the different intestinal sections (p < 0.05), whereas two-way ANOVA followed by a Šidák’s post hoc analysis (p < 0.05) was used for comparison of the endogenous surfactant content in mucus to the previously published content in the small intestinal lumen. All data were analyzed by ROUT (Q = 1%) to test for statistical outliers.

## Results and discussion

### Regional differences in the rheological properties of mucus

Due to the inter-animal and intra-sectional variations in the obtained volume of the mucus samples, it was not possible to obtain six replicates for all sections. For the distal jejunum section 2, only two rats had enough mucus in this section to analyze the rheological profile, while for the other sections, sufficient amounts of mucus were obtained from 4–6 rats (Fig. S1). In general, there seem to be a trend of less mucus in the distal jejunum section 2, as this was also prevalent in the rats used for the quantitative analyses.

**Fig. 1:**
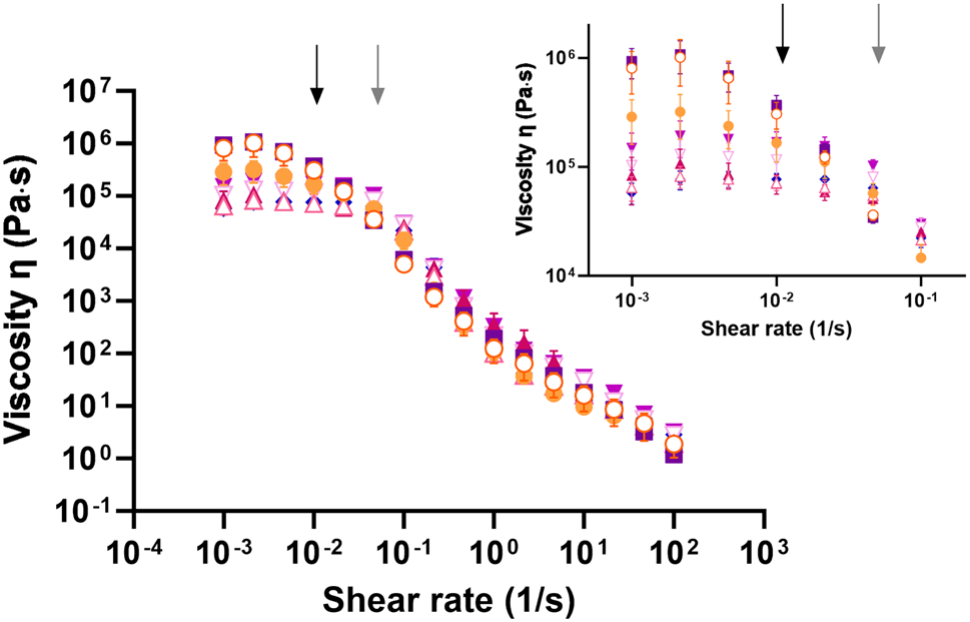
Viscosity as a function of shear rate of mucus from each of the eight sections of the rat small intestine, i.e., duodenum (  ), proximal jejunum section 1 (  ) and 2 (  ), mid jejunum section 1 (  ) and 2 (  ), distal jejunum section 1 (  ) and 2 (  ), and ileum (  ). Data are depicted as mean ± SEM (*n* = 2–6). The small window is a zoom of the lower shear rate region. The black arrows indicate the onset of the shear-thinning in the duodenum and proximal jejunum section 1, while the grey arrow indicates the area of onset for the other sections

As seen in Fig. 1, the mucus of the small intestine showed non-Newtonian shear-thinning properties with a plateau in the lower shear region followed by a shear thinning region. The rheological profiles for the individual rats in Fig. S1.

**Fig. 2:**
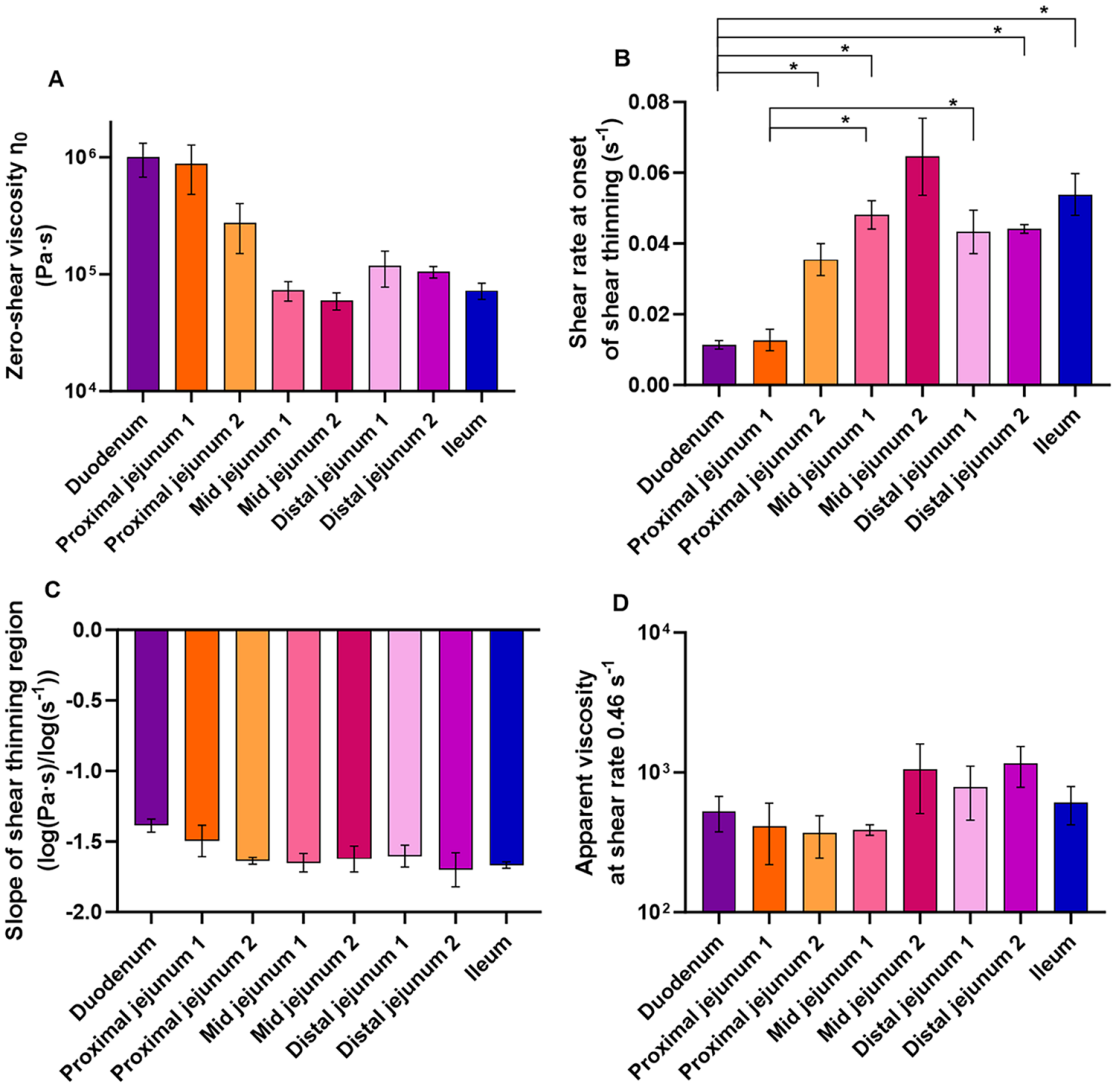
Rheological information from flow curves of the mucus from each of the eight sections of the rat small intestine: zero-shear rate viscosity (η_0_) (**A**), shear rate at the onset of shear-thinning (**B**), slope of the shear-thinning region (shear rates 0.03 – 10 s^−1^) (**C**), and apparent viscosity η_a_ at shear rate 0.46 s^-1^ (**D**). Data are depicted as mean ± SEM (*n* = 2–6). Statistically different values are marked with an asterisk when p < 0.05 according to a one-way ANOVA

The flow curves from the mucus samples obtained from the different sections of the small intestine can be divided into two groups according to their viscoelastic behavior at low shear rates, i.e., the flow curves of the duodenum and proximal jejunum section 1 displayed a higher viscosity than the other six sections (proximal jejunum 2 to ileum). This difference is more easily observed in the zoom of the lower shear rate region in Fig. 1 and upon a more detailed analysis of the flow curves, in which regional differences are observed for the zero rate viscosity and at the onset of the shear-thinning region (Fig. 2A-B). The mucus from the duodenum and proximal jejunum section 1 tended to have higher zero-shear viscosity (~ 9.4 · 10^5^ Pa·s) than mucus from the sections from mid jejunum 1 and onwards (~ 1.5 · 10^5^ Pa·s), albeit the difference was not statistically significant (Fig. 2A). Additionally, the mucus from the duodenum and proximal jejunum section 1 had significantly lower onsets of the shear-thinning region at a shear rate of ~ 0.01 s^−1^ compared to the onset in the range of 0.04–0.07 s^−1^ observed for the mucus of the other six sections (p < 0.05) (Figs. 1, 2B). There was no difference in the slopes of the shear-thinning region (shear rates 0.03 – 10 s^-1^) when comparing the mucus collected from the different sections of the small intestine (Fig. 2C). Similarly, there was no difference in the apparent viscosity of the mucus from various sections at the shear rate of 0.46 s^-1^ (Fig. 2D), which is the closest measuring point to the reported physiological shear rate near the duodenal wall in the rat (0.21–0.41 s^-1^) [24].

**Fig. 3:**
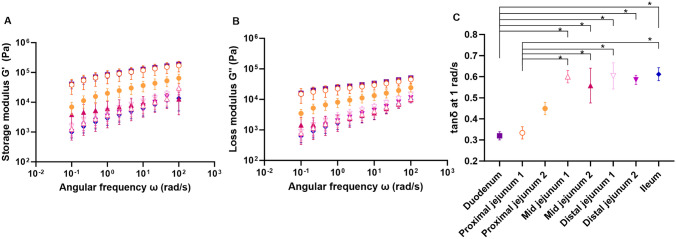
Comparison of the storage modulus G′ (**A**) and loss modulus G″ (**B**) from a frequency sweep of each of the eight sections of the rat small intestine, i.e., duodenum (  ), proximal jejunum section 1 (  ) and 2 (  ), mid jejunum section 1 (  ) and 2 (  ), distal jejunum section 1 (  ) and 2 (  ), and ileum (  ). The tan δ in the different sections of the rat small intestine at an angular frequency of 1 rad/s (**C**) was compared using a one-way ANOVA. Statistically different values are marked with an asterisk when p < 0.05. Data are depicted as mean ± SEM (*n* = 2–6)

The mucus layer of the small intestine proved to be a viscoelastic gel with solid-like properties as all sections had a higher G′ than G″ in the tested frequency range (Fig. 3A, B). This property is also evident from tan δ depicted in Fig. 3C, in which all sections showed solid-like behavior with tan δ < 1. As was the case with the shear flow viscosity, the moduli of the mucus from the first two sections, the duodenum and proximal jejunum section 1, were higher than the moduli of the mucus from the other six sections (Fig. 3A, B). Furthermore, tan δ was significantly lower for the first two sections at ~ 0.3 compared to the last five sections with tan δ ~ 0.6 (p < 0.05) (Fig. 3C). While still predominantly solid-like, as tan δ < 1, the mucus layer of the mid jejunum and onwards trended towards a less solid-like structure compared to the mucus of the duodenum and proximal jejunum section 1. An increase in tan δ might indicate a less rigid gel network in the lower sections of the small intestine compared to the upper sections, as has previously been observed in studies of porcine gastric mucus and mucoadhesive polymers [25].

The flow curves obtained in the current study are comparable to the flow curves presented in a study by Zahm et al., in which gastric and duodenal mucus obtained from fasted Wistar rats was studied [9]. Although the previous study observed no zero-shear plateau, possibly due to analysis at higher shear rates (1·10^-2^—5·10^1^ s^-1^), the viscosity of the duodenal mucus was in a similar range as reported in the current study (starting at 10^4^ – 10^6^ Pa·s). The slope of the shear-thinning region reported by Zahm et al. was, however, less steep at -0.82 to -0.93 log(Pa·s)/log(s^-1^) [9] when compared to the current study reporting a mean slope of -1.39 ± 0.04 log(Pa·s)/log(s^-1^) (Fig. 2C). Whether this difference is caused by experimental settings, or strain differences, is unclear. Boegh et al. studied the rheological behavior of porcine small intestinal mucus using analytical conditions similar to those used in the current study [7] and did not observe a zero-shear viscosity plateau, but a linear shear-thinning region starting at a comparatively lower viscosity of ~ 10^3^ Pa·s [7]. Canine mucus has been shown to have an even lower viscosity, starting the linear shear-thinning at a viscosity of ~ 10^2^ Pa·s [8]. It has to be noted that the rat mucus were analyzed at 25 °C (in the current study and in Zahm et al. [9]), whereas the porcine and canine mucus were analyzed at 37 °C [7, 8]. While the difference in experimental temperature would influence the results, it is not likely that this has caused the large differences observed (from 10^4^ – 10^6^ Pa·s in the rat to ~ 10^3^ Pa·s in pigs and ~ 10^2^ Pa·s in dogs), and the primary cause is thus expected to be differences in the rheological properties of the small intestinal mucus layer in the different species.

Similar to the rheological flow curves, the G′ of rat intestinal mucus (G′ at ~ 10^3^-10^5^ Pa; Fig. 3A) was generally higher than what has been reported in pigs (G′ at ~ 10^1^–10^2^ Pa [6, 26–28]) and dogs (G′ ~ 10^1^-10^2^ Pa [8]). While the tan δ of porcine duodenal mucus was lower than the rat duodenal mucus (0.15-0.16 [11, 28] vs. 0.3), studies have observed an increase in tan δ to 0.3–0.6 for porcine small intestinal mucus [10, 27, 28], which is similar to what was observed in the current study (Fig. 3C). Although the moduli values of rats are higher than those of other species, the tan δ seem to change in a similar pattern along the small intestine, suggesting that they have a similar gel network.

### Regional differences in mucus pH and concentrations of proteins and endogenous surfactants

**Fig. 4:**
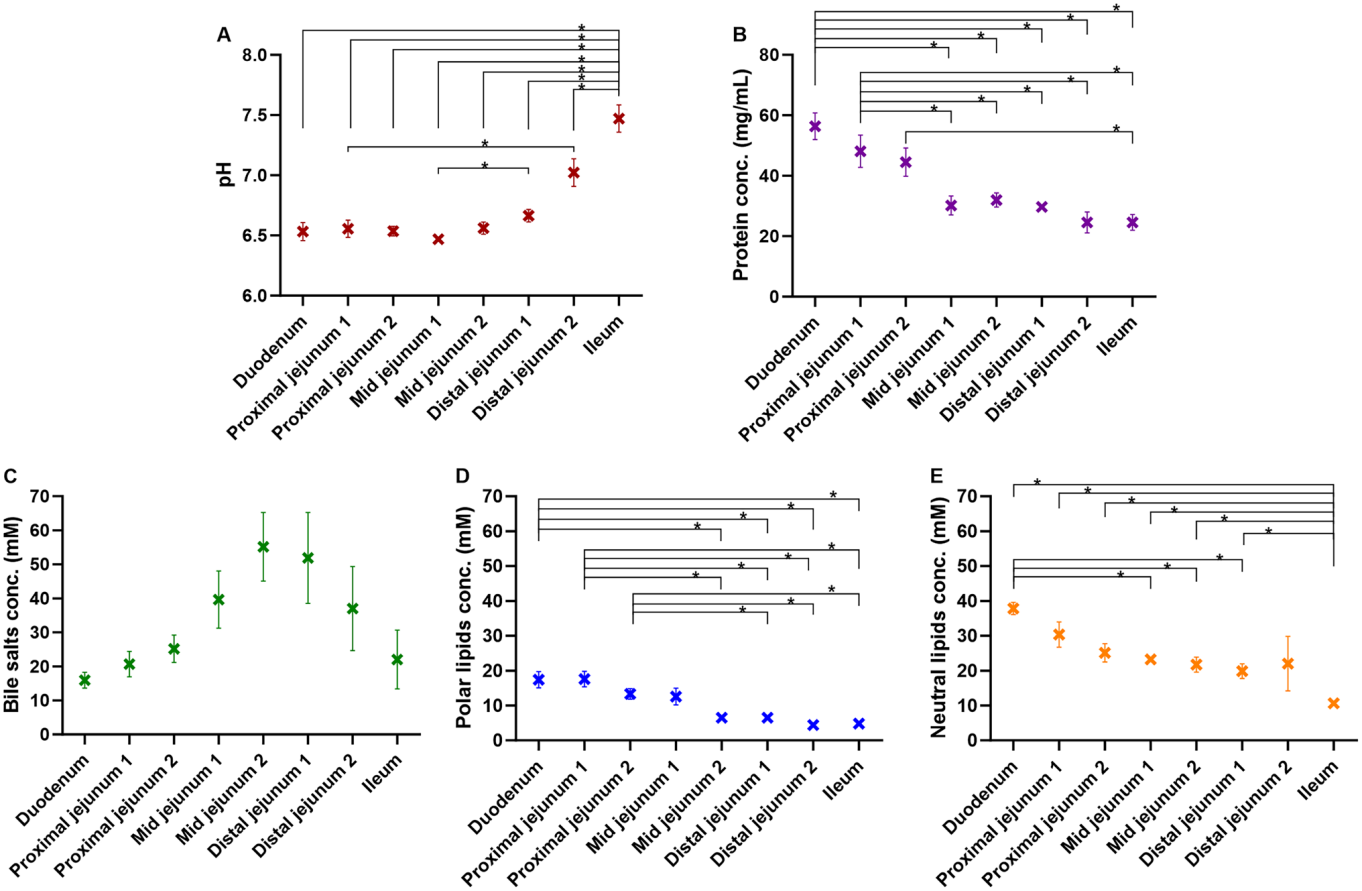
Mucus characteristics exhibiting regional differences in pH (**A**) and concentrations of proteins (**B**), bile salts (**C**), polar lipids (**D**), and neutral lipids (**E**) in the mucus of the eight sections of the rat small intestine. Data depicted as mean ± SEM (*n* = 4–8). Statistically different values are marked with an asterisk when p < 0.05 according to a one-way ANOVA

The mean values for the rat mucus pH and concentrations of proteins and endogenous surfactants are depicted in Fig. 4. The boxplots in Fig. S2 show the values of the individual rats.

A recent study by Klitgaard et al determined the regional differences in luminal pH and concentrations of endogenous surfactants in the rat small intestine [20]. The median placement of each of the four luminal incisions in the small intestine, made in the previous study, were paired with the corresponding mucus section in the current study, allowing a direct comparison between luminal and mucus values. Specifically, the previous study’s luminal incisions in the duodenum, proximal jejunum, mid jejunum, and ileum correspond to the mucus sections termed duodenum, proximal jejunum 1, mid jejunum 2, and ileum in the current study, respectively.

#### Mucus pH

In Fig. 4A, it is evident that the pH in the mucus layer was almost constant at ~ 6.5 from the duodenum to the distal jejunum 1, after which it steadily increased until reaching pH 7.5 ± 0.1 in the ileum. Only a few other studies have previously determined the pH of the mucus layer of the rat small intestine, often designated as the unstirred water layer, to which the mucus layer is indistinguishable. In agreement, Högerle and Winne studied the pH of the mucus layer during perfusion of different buffer systems in the proximal jejunum of fasted Wistar rats and found that the pH was largely stable at ~ 6.6 [29]. Lucas et al. and Daniel et al. both measured the pH of the mucus layer ex vivo from fed Wistar rats and found it to be pH 5.5–6.3 [30] and 6.8 ± 0.2 [31], respectively. Although there were differences in measurement methods, rat strains, and the prandial state of the rats, the values of the previous studies correlate very well with those of the present study.

Compared to the luminal pH in the rat small intestine [20], the mucus pH was significantly lower in both the duodenum (pH 6.53 ± 0.07 vs 6.92 ± 0.06 [20], (p = 0.006)) and the ileum (pH 7.47 ± 0.10 vs 7.87 ± 0.09 [20] (p = 0.006)). However, there were no significant differences between the mucus and luminal values for the proximal and mid jejunal pH.

#### Mucus protein concentrations

The protein concentration in the mucus layer decreased throughout the small intestine, starting at 56.4 ± 4.1 mg/mL in the duodenum and ending at 24.6 ± 2.4 mg/mL in the ileum (Fig. 4B). This decrease was statistically significant when comparing the protein concentration of the first two sections with that of each of the last five sections (p < 0.02). The change in protein concentration might, in part, be due to differences in mucin concentrations, as the colorimetric quantification kit would also react to the protein backbone of the mucins and not just non-mucin proteins such as serum albumin present in the sample. Previous studies have reported 31.8-50 mg/mL mucin in the duodenum and 20-30 mg/mL in the small intestine of pigs [4, 11, 32], indicating a decrease in overall mucin concentration. These studies have, however, used different methods of quantification and treatment of the mucus samples, making a comparison to the current study difficult. In a recent study on Wistar rats, the expression of mucin subtype Muc5ac was shown to drastically decrease from the duodenum to the jejunum [33], which has also been observed in pigs [6] and dogs [8]. However, how much the mucins in the rat mucus contributed to the total protein concentration in the mucus is unknown, and further studies are needed to assess the concentration of mucin in rat intestinal mucus. Recently, Mortensen et al. determined the total protein concentration of porcine mucus from the proximal jejunum, using a similar protein assay kit as the one used in the current study, and found a protein concentration of 126 ± 18 mg/mL [26]. Therefore, by comparison to this recent study and approximation to the others studies on pig mucus [5, 26], it seems that there might be a lower concentration of proteins in the mucus from fasted rats.

#### Mucus bile salt concentrations

The individual bile salts identified in the mucus of the small intestine of the rat were tauro-β-muricholic acid, taurocholic acid, glycocholic acid, taurochenodeoxycholic acid, taurodeoxycholic acid, and cholic acid, with tauro-beta-muricholic acid and taurocholic acid being the most prominent (Fig. S3A). The total concentration of bile salts was 16.0 ± 2.2 mM in the duodenal mucus and gradually increased until peaking in the second section of the mid jejunum (55.1 ± 9.5 mM), after which it steadily decreased until the ileum (22.0 ± 8.1 mM) (Fig. 4C). Curiously, some sections in some rats contained no bile salts (Fig. S2C), even though the neighboring sections in the same rat had bile salts, and the other endogenous surfactants were recovered from the mucus of that section. This observation contributed to the high inter-individual variability observed for the bile salt concentrations in the mucus layer (Figs. 4 and S2C).

Although no significant difference in mucus bile salt concentrations was observed between the different sections of the small intestine (Fig. 4C), there is a clear pattern that follows the same tendency as the luminal bile salt concentrations [20]; i.e., increasing bile salt concentrations until the mid-jejunum and then a decrease, presumably caused by active bile salt reabsorption in the latter half of the small intestine [34]. The concentrations of bile salts in the mucus layer were significantly lower in the proximal jejunum (20.7 ± 3.5 mM) and mid jejunum (55.1 ± 9.5 mM) when compared to the luminal values (100.0 ± 10.2 mM and 123.4 ± 6.6 mM [20]). In the duodenum and ileum there were no significant differences between the mucus and luminal values. However, albeit it was not significantly different, there was a trend towards higher concentrations of bile salts in the mucus layer of the ileum (22.0 ± 8.1 mM) when compared to the luminal fluids (4.3 ± 2.2 mM [20]).

This is the first time the bile salt concentration in the mucus layer of the rat small intestine has been quantified. Since the rat lacks a gallbladder, bile salts and other endogenous surfactants are excreted to the small intestine at a continuous rate, which is likely the cause of the high endogenous concentrations of bile salts in the small intestine, including the mucus layer [35].

#### Mucus polar lipid concentrations

The total polar lipid concentration was highest in the mucus of the upper half of the small intestine (~ 15 mM) and decreased to ~ 5 mM in the latter half, from the second mid jejunal section and onwards (Fig. 4D). The most prominent polar lipids were lysophosphatidylcholine and phosphatidylcholine, with a minor contribution to the total polar lipid concentration from phosphatidylethanolamine and barely any contribution from sphingomyelin (Fig. S3B). In the upper half of the small intestine, the contribution to the total polar lipid concentration was almost evenly divided between lysophosphatidylcholine and phosphatidylcholine. There was, however, a steady and gradual decrease in the concentration of lysophosphatidylcholine, that was highest in the duodenum (7.9 ± 1.7 mM) and barely detectable in the last section of the small intestine (0.1 ± 0.1 mM) (Fig. S3B).

Compared to the luminal values [20], there was a significantly higher concentration of polar lipids in the mucus layer of the duodenum (17.4 ± 2.2 mM vs. 7.3 ± 1.1 mM [20] (p = 0.002)). However, the polar lipid concentration in the following proximal jejunum section was significantly lower in the mucus layer compared to that of the lumen (17.6 ± 2.1 mM vs. 24.9 ± 3.6 mM [20] (p = 0.049)). There were no significant difference between the mucus and luminal fluid concentrations of polar lipids in the lower half of the small intestine.

In porcine mucus from the proximal small intestine, Larhed et al. determined that lipids contributed to 37% (w/w) of the dry weight [5]. The authors further determined that 5.1% (w/w) of the total amount of lipids recovered were polar lipids, primarily consisting of lysophosphatidylcholine (2.7% (w/w)) and to a lesser degree of phosphatidylethanolamine (0.9% (w/w)), phosphatidylcholine (0.8% (w/w)), and sphingomyelin (0.7% (w/w)), whereas the remaining lipids were neutral lipids [5].

#### Mucus neutral lipid concentrations

The neutral lipids present in the intestinal mucus layer of rats were identified as linoleic acid, oleic acid, palmitic acid, stearic acid, and cholesterol, with linoleic acid as the main contributor to the total neutral lipid concentration (Fig. S3C). There was a steady, gradual decrease in the concentration of neutral lipids throughout the small intestine, starting at 37.8 ± 1.6 mM in the mucus of the duodenum and ending at 10.7 ± 1.1 mM in the ileum (Fig. 4E). The observed decrease in total neutral lipid concentration (Fig. 4E) seemed to be dominated primarily by the concentration of linoleic acid, as the concentrations of the other neutral lipids only had a slight decrease from ~ 5 mM in the duodenum to ~ 3 mM in the ileum (Fig. S3C).

In comparison to the luminal concentrations in the rat, the neutral lipid concentrations in the mucus layer were significantly higher than those of the lumen in the duodenum (37.8 ± 1.6 mM vs 15.1 ± 1.2 mM [20] (p < 0.0001)), proximal jejunum (30.4 ± 3.4 mM vs 11.3 ± 0.5 mM [20] (p < 0.0001)), and mid jejunum (21.7 ± 2.0 mM vs 8.3 ± 1.9 mM [20], (p = 0.0005)). Though trending towards higher values, the mucus concentrations in the ileum were not significantly higher than the luminal concentrations (10.7 ± 1.1 mM vs 4.6 ± 1.4 mM [20]).

Larhed et al. described a similarly high amount of linoleic acid in the porcine mucus layer in the proximal small intestine. Of the lipids determined by Larhed et al, the majority were designated as neutral lipids with 24% (w/w) linoleic acid, while other free fatty acids each contributed with 13-17.7% (w/w) and cholesterol with 12% of the total lipid content [5]. The composition of neutral lipids was like those of the current study in rat mucus, albeit the rat had a slightly higher ratio of linoleic acid (Fig. S3C). Overall, the ratio of neutral to polar lipids reported for pigs are higher than those determined in the current study in the rat small intestinal mucus, which could be due to higher polar lipid concentrations in the rat luminal fluids [5, 20, 36].

### Relation between rheological properties and concentrations of proteins and lipids

**Fig. 5:**
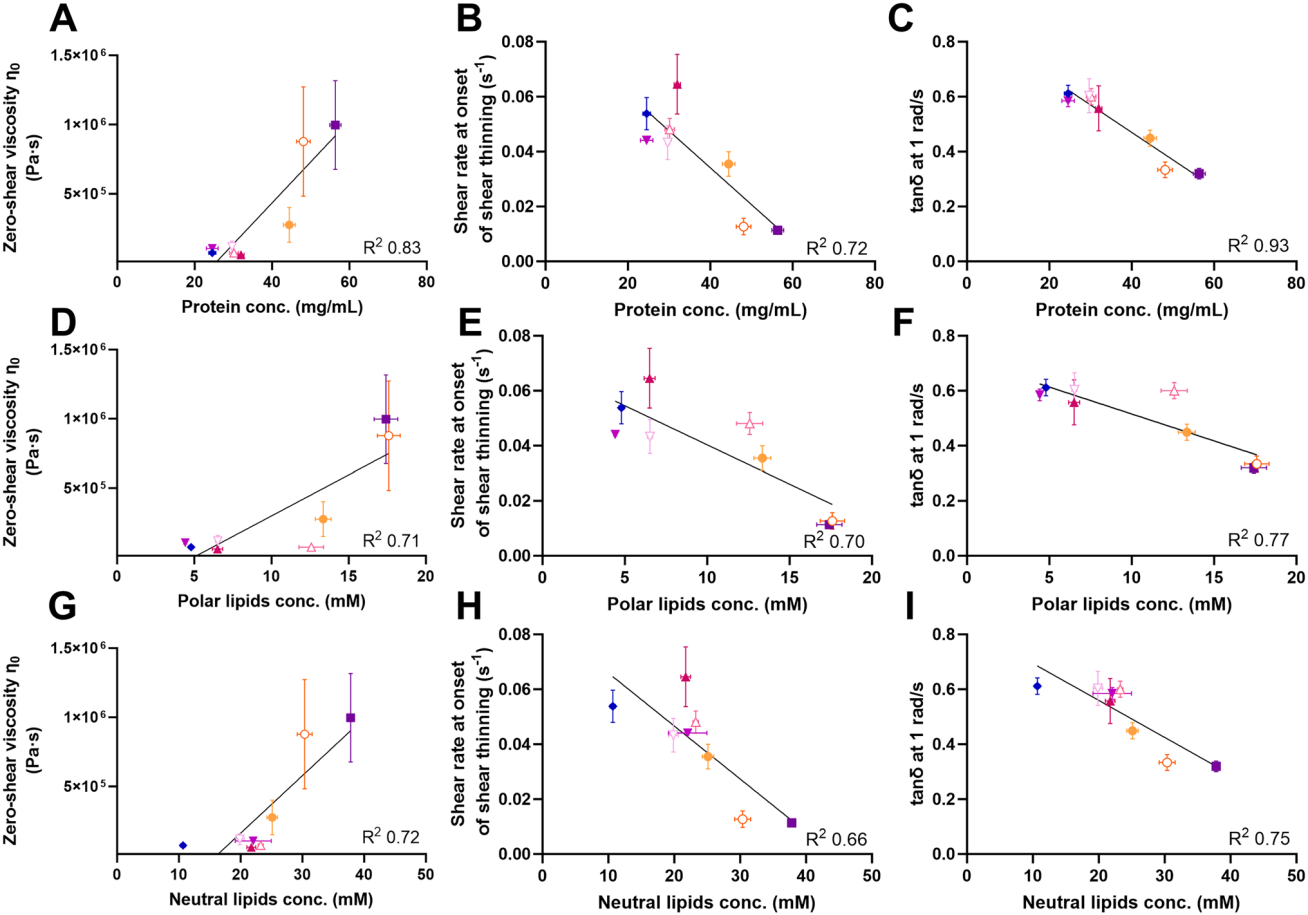
Relations between the concentrations of proteins (**A**-**C**), polar lipids (**D**-**F**) and neutral lipids (**G**-**I**) and the rheological properties, i.e. the zero-shear rate viscosity (η_0_) (**A**, **D**, **G**), shear rate at the onset of shear-thinning (**B**, **E**, **H**), and tan δ at an angular frequency of 1 rad/s (**C**, **F**, **I**) of each of the eight sections of the rat small intestine. Duodenum (  ), proximal jejunum section 1 (  ) and 2 (  ), mid jejunum section 1 (  ) and 2 (  ), distal jejunum section 1 (  ) and 2 (  ), and ileum (  ). Data are depicted as mean ± SEM (n = 2–8)

There seems to be an integral interplay between the endogenous concentrations of proteins and the rheological properties of the small intestinal mucus layer of the rat. The observed regional differences in the rheological properties of the mucus layer coincided with the regional differences in mucus protein concentration, i.e., the first two sections of the small intestine displaying a more viscous mucus (Figs. 1, 3) were also the sections containing the highest amount of proteins (Fig. 4B). Similarly, the remaining sections that showed similar rheological profiles had similar protein concentrations. This is also evident in Fig. 5, in which the higher protein concentrations in the duodenum and proximal jejunum section 1 coincides with a higher zero-shear viscosity, lower shear rate for onset of shear thinning, and lower tan δ when compared to the lower sections (Fig. 5A-C).

Previous studies have reported on the importance of polar and neutral lipids in the mucus to mimic the rheological properties and structure of the mucus network [5, 7]. The observed decrease in both polar and neutral lipid concentration could, therefore, further explain the change in rheological properties and decreased rigidity of the gel network when comparing mucus from the upper to lower part of the small intestine (Figs. 3C, 4D, E, 5D-I). While only the comparison between tan δ and the protein concentration (Fig. 5C) provided a linear relation of R^2^ > 0.9, there is a clear overall trend between the rheological properties and the mucus characteristics, i.e., the high concentrations of proteins and lipids in the upper small intestine relates to high zero-shear viscosity, early onset of shear thinning and a lower tan δ (Fig. 5). Therefore, while the mucins are a dominant cause of the viscoelastic behavior of mucus, the other components such as proteins and lipids also play an essential role in creating the viscoelastic gel structure of the mucus.

### Mucus affecting drug absorption

Mucus is commonly seen as a barrier to drug absorption, in part due to its viscoelastic properties slowing the diffusion of drugs particles and larger drug molecules [3, 4, 37]. However, considering the findings of the current study and the fact that the small intestinal mucus fluid volume is approximately five times higher than the corresponding luminal fluid volume [38], mucus might pay a more significant role in drug absorption than hitherto expected. The presence of the endogenous surfactants in the mucus layer could aid in the solubilization of orally administered poorly water-soluble drugs as the conditions of the small intestine are known to affect the solubility and dissolution of poorly water-soluble drugs and thus, potentially, the transport across the epithelial membrane [38–42]. The pH in the small intestine, both luminal and in the mucus, will affect ionizable poorly water-soluble drugs and controlled release drug delivery systems relying on a specific pH. The pH can thus affect drug solubility and diffusivity, since positively charged drugs will have a slower diffusion through the mucus layer compared to neutral or negatively charged drugs [3]. Changes in pH would be most prominent in the duodenum and ileum, as the difference between luminal and mucus pH was not significant in the other sections of the small intestine of the rat.

The concentrations of the individual type of endogenous surfactants has previously been shown to affect the solubility and dissolution of poorly water-soluble drugs to different degrees, in which one drug might primarily be affected by the bile salt concentration, whereas others depend on the lipid concentrations or the ratio between these surfactants [43]. Overall, the concentrations of bile salts in all sections of the small intestinal mucus exceed that of the reported critical micellar concentrations of 2–20 µM, which is lowered when forming mixed micelles with lipids [44]. Poorly water-soluble drugs have to be solubilized, either molecularly dispersed in the free form or in vesicles or micelles, and traverse the mucus layer before permeating the epithelial layer as free dissolved drug [16, 45]. These vesicles and micelles are theorized to shuttle the drug across the mucus layer, dissociate and thus create a supersaturated reservoir of molecularly dissolved drug in the mucus close to the epithelial wall [45, 46].

Yeap et al. showed some of these beneficial properties of mucus on drug absorption when performing in vitro tests on two poorly water-soluble drugs [47]. They reported that the presence of diluted porcine small intestinal mucus improved the solubilization of two poorly water-soluble drugs in vitro*,* compared to commercially available porcine gastric mucin, attributing this improvement to the presence of lipids and proteins in the native mucus. The presence of mucin (from either source) improved the supersaturation stabilization of both drugs, and when testing the permeation of one supersaturated poorly water-soluble drug, Yeap et al. observed a flux enhancement in presence of mucus producing co-cultures compared to Caco-2 monolayers [47]. The study by Yeap et al. emphasizes the potential of incorporating mucus in in vitro assessment as the improved drug solubilization, supersaturation stabilization and absorption due to the presence of mucus is expected to have a similar influence on drugs in vivo. The properties of rat small intestinal mucus described in the current study should enable future studies to include the influence of mucus in the preclinical assessment of drugs and implement mucus in the in vitro evaluation of drugs for improved understanding of the oral absorption of drug delivery systems in rats.

## Conclusion

To the best of the authors’ knowledge, this is the first study to characterize interregional differences in the rheological properties and composition of rat small intestinal mucus. The rat small intestinal mucus was found to be a non-Newtonian, viscoelastic gel that changes rheological properties and composition dependent on the small intestine region. The upper two sections of the small intestinal mucus differ from the rest of the small intestine in the rheological profile with higher zero-shear viscosity, lower onset of shear thinning region and lower tan δ as well as higher protein concentrations. The presence and concentration of bile salts, polar lipids, and neutral lipids at levels above the critical micellar concentration indicates that the mucus layer has the potential to solubilize poorly water-soluble drugs and thereby facilitate absorption, rather than just being a barrier to absorption. Thus, the current study adds to the information on the rat small intestinal mucus and its role in drug absorption. The data can be used for improving the in vitro models for assessment of oral drug delivery systems and lead to improved and better drugs on the market.

## Supplementary Information

Below is the link to the electronic supplementary material.

Supplementary file1 (DOCX 867 KB)

## Data Availability

Not applicable.

## Abbreviation

RP-HPLC-CAD: Reverse-phase high-performance liquid chromatography with charged aerosol detection
