# Supplementary material for: Characterizing interregional differences in the rheological properties and composition of rat small intestinal mucus
Source: Drug Deliv Transl Res. 2024 Mar 25;14(11):3309–20. doi: 10.1007/s13346-024-01574-1 (PMC11445339; doi:10.1007/s13346-024-01574-1)
Supplement: Supplementary file 1 — Supplementary file1 (DOCX 867 KB) [file 13346_2024_1574_MOESM1_ESM.docx]

**Supplementary Information**


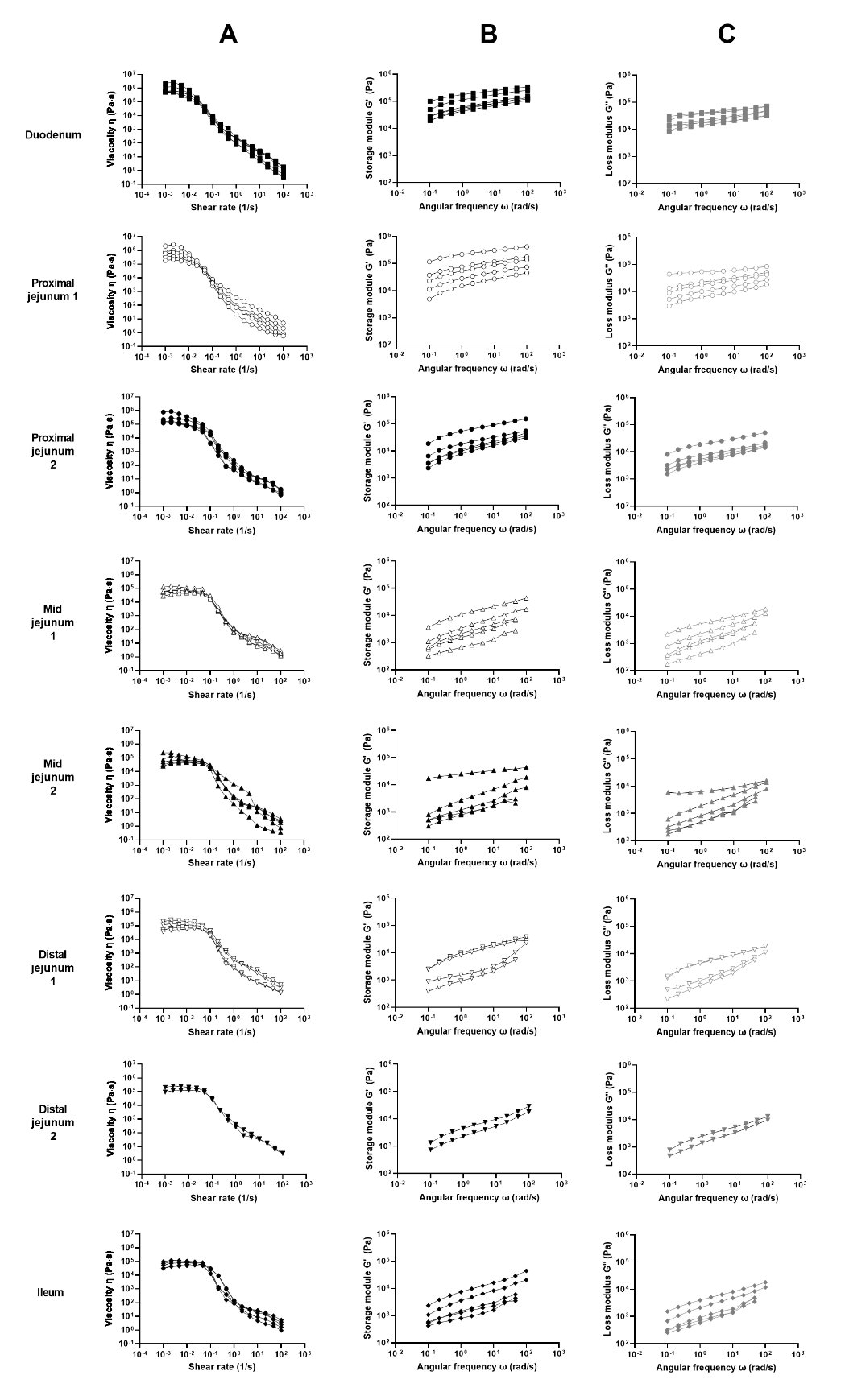


**Supplementary Figure S1.** Rheological properties of the small intestinal mucus divided into sections and depicting the viscosity as a function of shear (A), storage modulus G′ (B) and loss modulus G″ (C). Each curve represent the measurement from one rat in the respective section of the small intestine.


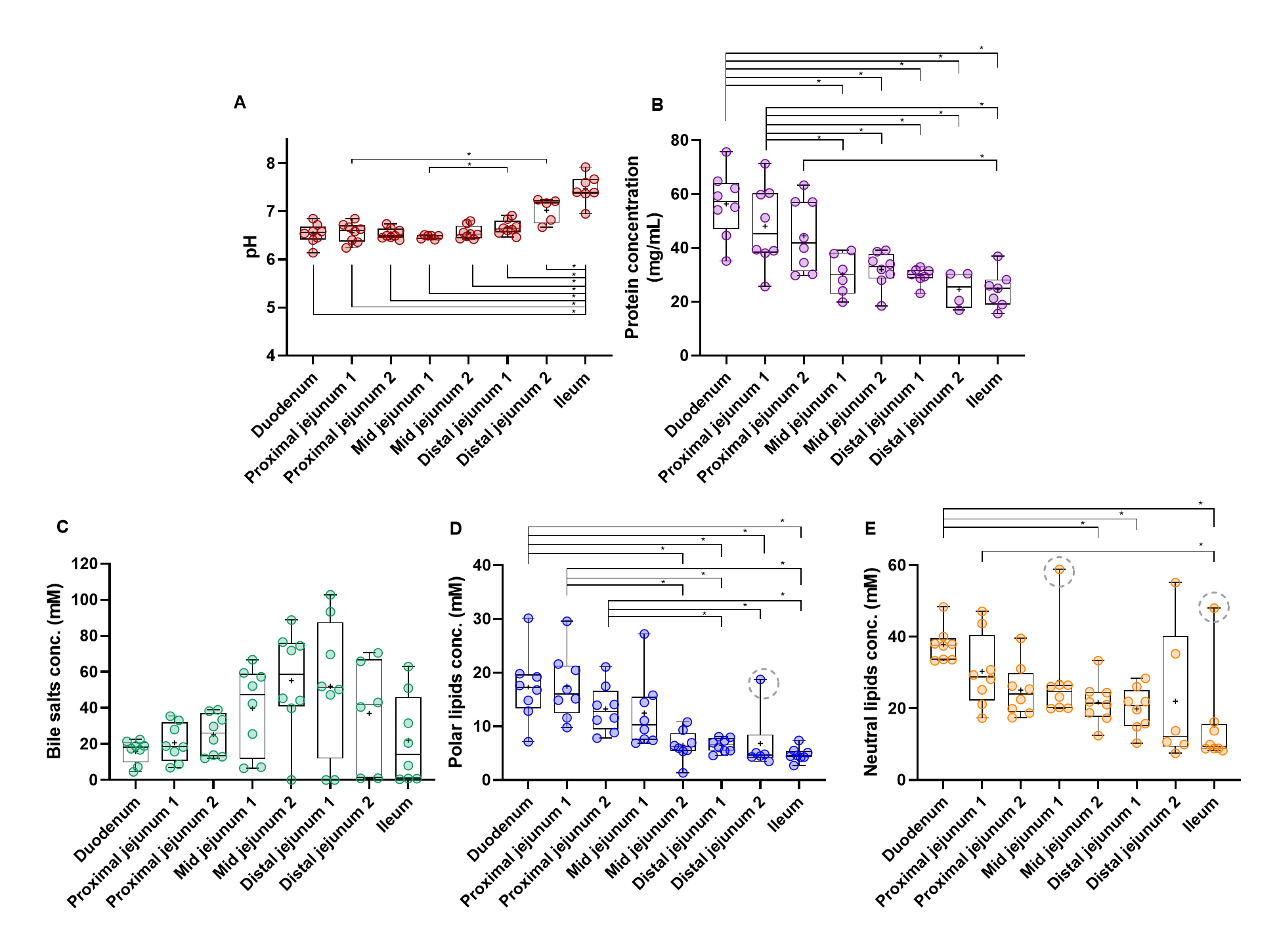


**Supplementary Figure S2.** Regional differences in pH (**A**) and concentrations of proteins (**B**), bile salts (**C**), polar lipids (**D**), and neutral lipids (**E**) in the mucus of the eight sections of the rat small intestine. The boxplots show the individual values, median (line), mean (+), minimum and maximum values as well as the 25%- and 75%-percentiles. Significantly different values have been marked with an asterisk where *p* < 0.05. Values that were deemed as statistical outliers by ROUT testing have been indicated by a dotted circle.


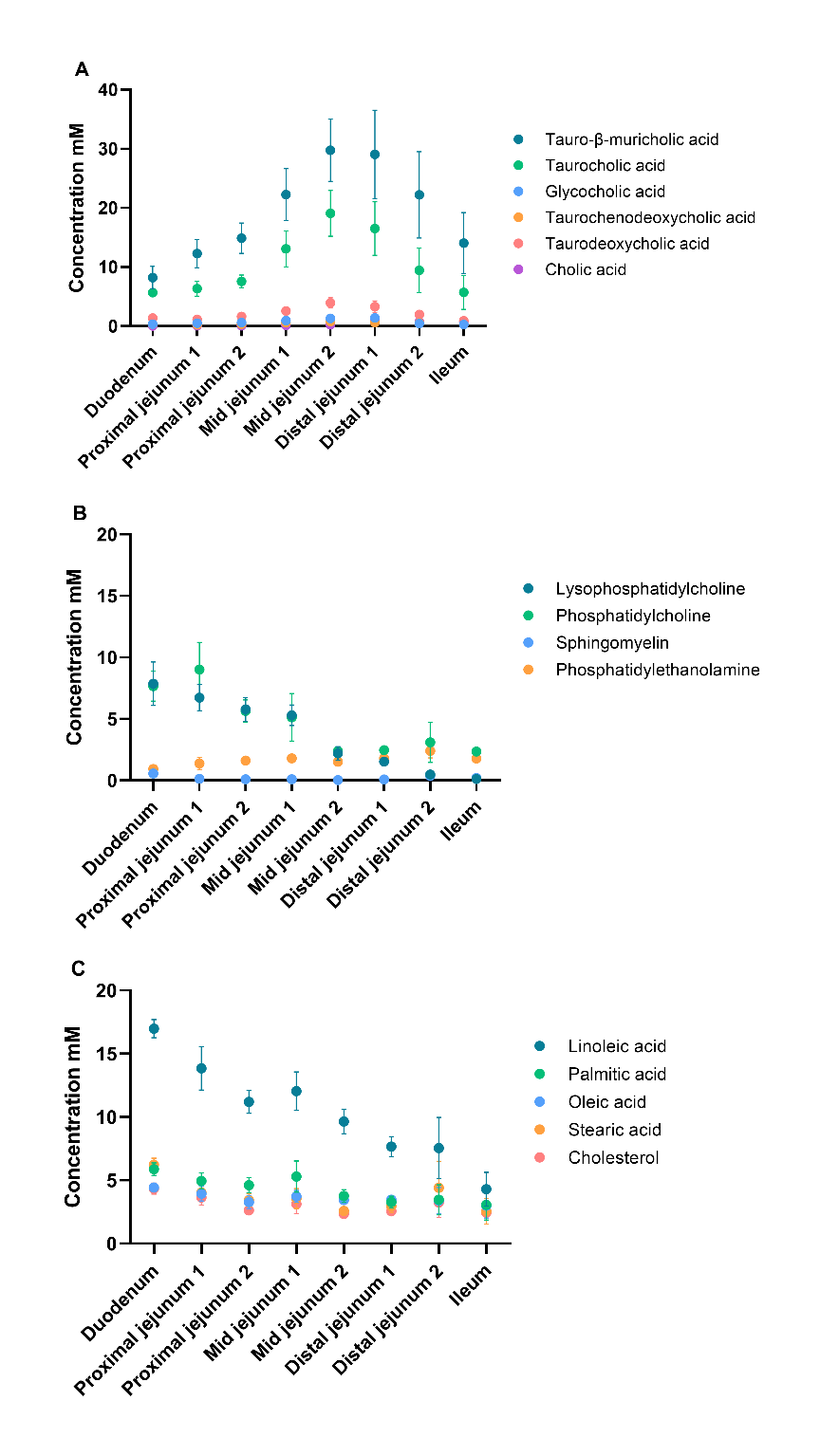


**Supplementary Figure S3.** Regional differences in concentrations of individual bile salts (A), polar lipids (B), and neutral lipids (C) in the mucus of the eight sections of the rat small intestine. Data depicted as mean ± SEM (*n*=4-8).
